# Overactivation of the complement system may be involved in intrarenal arteriolar lesions in IgA nephropathy

**DOI:** 10.3389/fmed.2022.945913

**Published:** 2022-08-03

**Authors:** Wei-yi Guo, Xiu-ping An, Li-jun Sun, Hong-rui Dong, Wen-rong Cheng, Nan Ye, Guo-qin Wang, Xiao-yi Xu, Zhi-rui Zhao, Hong Cheng

**Affiliations:** Renal Division, Department of Medicine, Beijing Anzhen Hospital, Capital Medical University, Beijing, China

**Keywords:** IgA nephrology, intrarenal arteriolar lesion, complement system, C3c, renal survival

## Abstract

**Introduction:**

IgA nephropathy (IgAN) encompasses a wide range of clinical and histology features. Some patients present without hematuria, with or without hypertension, still rapidly progress in renal function. Renal pathology of this part of patients were predominant intrarenal arteriolar lesions, rarely presented glomerular proliferative lesions. We aim to investigate the clinical and pathological characteristics and prognosis of these IgAN patients and initially explore whether the abnormal activation of complement is involved in the intrarenal arteriolar lesions of IgAN.

**Methods:**

A total of 866 patients with renal biopsy-proven IgAN diagnosed at Beijing Anzhen Hospital were recruited. IgAN patients without intrarenal arteriolar lesions and proliferative lesions were excluded (*n* = 115), the rest were divided into arteriolar lesions group (*n* = 202) and proliferative lesions group (*n* = 549). Among them, 255 patients were regularly followed up for at least 1 year. Renal biopsy tissues of 104 IgAN patients were stained for complement components by immunohistochemistry and immunofluorescence.

**Results:**

Compared with proliferative lesions group, the arteriolar lesions group experienced high percentage of hypertension (*p* = 0.004), low percentage of gross hematuria (*p* = 0.001), microscopic hematuria (*p* < 0.001) and less initial proteinuria (*p* = 0.033). Renal survival between the two groups was not significantly different (*p* = 0.133). MBL, C4d, FH and FHR5, C3c, and MAC deposited on intrarenal arteriole in arteriolar lesions group. Compare with the proliferative lesion group, the arteriolar lesions group exhibited a higher intensity of C3c deposition on the intrarenal arterioles (*p* = 0.048). C3c and CD31 co-deposited on intrarenal arterioles area in patients with intrarenal arteriolar lesions.

**Conclusion:**

Renal survival of the IgAN patients in arteriolar lesions group was not better than those in proliferative lesions group. Abnormal activation of complement may be involved in the pathogenesis of arteriolar damage through the injury of endothelial cells in this clinical phenotype of IgAN.

## Introduction

Immunoglobulin A nephropathy (IgAN) is the most common primary glomerulonephritis worldwide (1). It is a leading cause of chronic kidney disease and progresses to end-stage kidney disease approximately 20 years after diagnosis in up to 20–40% of patients (2). Patients with IgAN display considerable heterogeneity in clinical manifestations and pathological phenotypes (3). In addition to the typical clinical manifestations, such as recurrent episodes of gross hematuria during or immediately following mucosal infections (4), some patients also present without hematuria, with or without hypertension, and show a rapid decline in renal function. The renal pathology of this group of patients shows predominantly intrarenal arteriolar lesions, and seldom presents as glomerular proliferative lesions. At present, there is no unified understanding of the clinical manifestations, prognosis or pathogenesis of patients with IgAN with predominant intrarenal arteriolar lesions.

The role of intrarenal arteriolar lesions in the prognosis of IgAN remains under debate owing to inconsistent study results. For the treatment of this clinical phenotype dominated by intrarenal arteriolar lesions, clinical practice is to control blood pressure to reach the target. There remains a lack of guidance and recommendations for immunosuppressive therapy for these patients (5). Previous studies have shown that blood pressure is an independent factor that affects intrarenal arteriolar disease (6). However, we noticed that some IgAN patients with normal blood pressure also presented with severe intrarenal arteriolar damage (7–9), suggesting that in addition to blood pressure, other factors may be involved in the pathogenesis of intrarenal arteriolar lesions.

**FIGURE 1:**
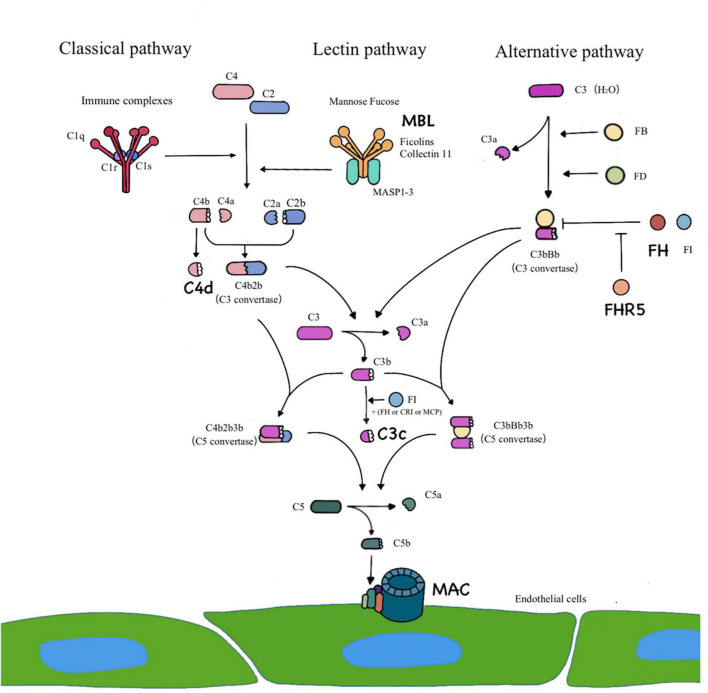
Three pathways of complement activation. The classical pathway is activated by IgG– and/or IgM–containing immune complexes. The lectin pathway requires a particular sugar moiety pattern to be recognized and bound by MBL/Ficolins/Collectin 11, leading to a classical pathway–like activation cascade. C4d represents the activation of classical pathway or/and lectin pathway. The alternative pathway is constantly initiated by spontaneous hydrolysis of C3 [C3b (H_2_O)] that is efficiently powered by the covalent attachment of C3b on an activating surface. Complement factor H (FH) and factor H related protein 5 (FHR5) are regulator proteins of alternative pathway. Three pathways lead to formation of C3 convertase and converge at the C3 level. The addition of C3b to the C3 convertase creates C5 convertase, which triggers the formation of the terminal pathway complete complex (C5b-9), which is also known as membrane attack complex.

Increasing evidence has implied the importance of alternative pathway- and/or lectin pathway-induced complement activation in IgAN (10). Complement activation can generally occur through three different pathways (Figure 1). In patients with IgAN, the components of complement activation have been commonly detected in the renal biopsy specimens, circulatory immune complexes, blood and urine (10–17). In recent years, several studies have showed that complement C4d is related to vascular lesions, especially thrombotic microvascular disease-like lesions (18). Arteriolar C4d is a potential biomarker for disease progression in IgAN (19, 20). Studies of atherosclerosis have revealed that sublytic C5b-9 assembly leads to the activation and proliferation of smooth muscle and endothelial cells (21). Previous studies on complement in vascular injury have focused mainly on thrombotic microangiopathy and atherosclerotic damage to the large and middle arteries. The role of complement system activation in intrarenal arteriosclerosis of IgAN is rarely discussed.

This study aims to investigate the clinical and pathological characteristics and prognosis of patients with IgAN with predominant arteriolar lesions and to initially explore whether the abnormal activation of complement is involved in the intrarenal arteriolar lesions of IgAN.

## Materials and methods

### Study population

**FIGURE 2:**
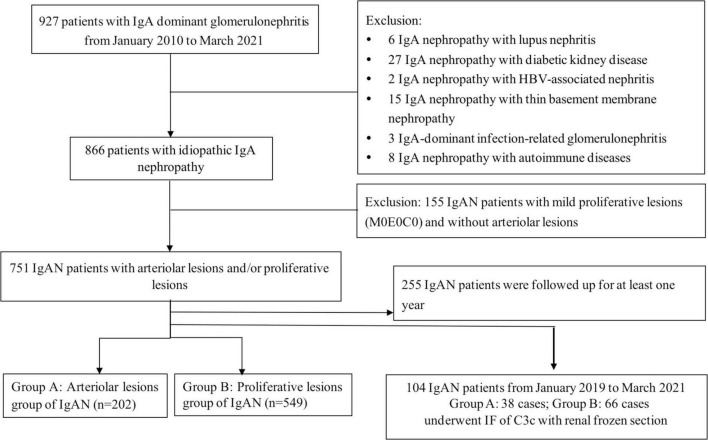
Flow diagram of patients of IgA nephropathy enrolled in the study. A total of 866 patients with renal biopsy-proven IgAN diagnosed from January 2010 to April 2021. IgAN patients without intrarenal arteriolar lesions and proliferative lesions were excluded (*n* = 115), the rest were divided into arteriolar lesions group (group A: *n* = 202) and proliferative lesions group (group B: *n* = 549). Among them, 255 patients were regularly followed up for at least 1 year. Among the enrolled patients (*n* = 751, group A and group B), frozen renal biopsies of 104 patients from January 2019 to March 2021 were enrolled for immunofluorescence of C3c.

From January 2010 to March 2021, the baseline data of 866 patients with renal biopsy-proven IgAN diagnosed at Beijing Anzhen Hospital were included in this study. The diagnosis of IgAN was based upon the presence of dominant IgA deposition in the mesangial area as detected by immunofluorescence and electron-dense material deposition in the mesangial area as observed with electron microscopy. Patients with IgA vasculitis, liver cirrhosis, systemic lupus and other secondary etiologies of IgAN identified by detailed clinical and laboratory examinations were excluded. IgAN patients without intrarenal arteriolar lesion and proliferative lesion were excluded (*n* = 115). Among the enrolled patients (*n* = 751), frozen renal biopsies of 104 patients from January 2019 to March 2021 were enrolled for immunofluorescence of C3c. A flow diagram is shown in Figure 2.

The research was conducted in compliance with the principles of the Declaration of Helsinki and approved by the ethics committees of Beijing Anzhen Hospital. Informed consent was obtained from all enrolled individuals.

### Clinical manifestations

Clinical manifestations at the time of renal biopsy were collected from the medical records. High blood pressure (HBP) was defined as a systolic blood pressure (SBP) of 130 mmHg or more, a diastolic blood pressure (DBP) of 80 mmHg or more, or the use of antihypertensive medications to prevent hypertension. Hematuria was defined as gross hematuria or microscopic hematuria (RBC > 3/HP, with microscopic examination of sediment after centrifugation). The glomerular filtration rate (GFR) of IgAN patients was calculated using the Modified Glomerular Filtration Rate Estimating Equation (22).

### Histological manifestations

Renal biopsy specimens were processed routinely for light, immunofluorescence and electron microscopy. Processing for light microscopy examination included hematoxylin and eosin, periodic acid-Schiff, periodic acid-silver methenamine, and Masson’s trichrome (PAM-Masson) staining. Immunofluorescence staining for IgG, IgA, IgM, C3, C1q, and FRA was routinely performed. Intrarenal arterial lesions include intrarenal arterial arteriolar wall thickening, arterial occlusion, arterial wall sclerosis and hyaline change. Arterial arteriolar wall thickening was defined when the cross-sectional ratio of the luminal diameter to the outer diameter was less than approximately 0.48, according to our previous study (23). Hyaline change was defined as a glassy, pink or red eosinophilic homogenous appearance of the arteriolar wall without cell proliferation.

**FIGURE 3:**
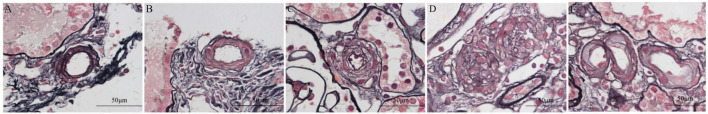
Periodic acid-silver methenamine and Masson’s trichrome (PAM-Masson) staining of intrarenal arterioles in patients with immunoglobulin A nephropathy (IgAN). Wall thickening was scored based on over 50% of intrarenal arteries and arterioles being affected relative to the total number of all arterial cross-sections, and the ratio of the luminal diameter to the outer diameter was as follows: 0 (> 0.48) [0.575, **(A)**], 1 (0.45∼0.48) [0.479, **(B)**], 2 (<0.45) [0.253, **(C)**], and 3 (0, arterial occlusion) [0, **(D)**]. Hyaline changes **(E)** (Original magnification × 1,000).

Wall thickening was scored based on over 50% of intrarenal arteries and arterioles being affected relative to the total number of all arterial cross-sections, and the ratio of the luminal diameter to the outer diameter was as follows: 0 (> 0.48) (Figure 3A), 1 (0.45∼0.48) (Figure 3B), 2 (< 0.45) (Figure 3C), and 3 (0, arterial occlusion) (Figure 3D). Hyaline changes (Figure 3E) were scored based on the percentage of affected intrarenal arteries and arterioles relative to the total number of all arterial cross-sections: 0, no hyaline; 1, intrarenal arterial lesion involvement < 25%; 2, intrarenal arterial lesion involvement > 25%. The sum score of all changes was used to grade intrarenal arterial lesions as mild (1), moderate (2–3), or severe (4–5) based on the total of the score of the ratio of the luminal diameter to the outer diameter and Hyaline changes.

The Oxford classification and the intrarenal arterial lesions score was used to evaluate pathological lesions for individuals with more than eight glomeruli and more than eight intrarenal arteries and arterioles in biopsy specimens (24). Kidney biopsies from all patients were reviewed independently by Wei-yi Guo and Li-jun Sun who were blinded to clinical data.

Patients without intrarenal arteriolar lesions and proliferative lesions were excluded (*n* = 115), and the rest were divided into two groups based on the main histological lesion: the arteriolar lesions group (group A) (*n* = 202), including patients with predominant intrarenal arterial lesions with mild Oxford mesangial hypercellularity, and without endocapillary hypercellularity or crescents (M0E0C0); the proliferative lesions group (group B) (*n* = 549), which consisted of patients with glomerular predominant proliferative lesions including Oxford mesangial hypercellularity and/or endocapillary hypercellularity and/or crescents (M1 and/or E1 and/or C1/2) with or without intrarenal arterial lesions. Among these patients, 68 patients in group A and 187 patients in group B were regularly followed up for at least 1 year, or reaching end-stage renal disease (ESRD) within 12 months.

### Outcomes

The composite outcome was defined as a 30% decline in eGFR, ESRD (eGFR < 15 ml/min/1.73 m^2^, the requirement for maintenance dialysis for at least 6 months, or kidney transplantation) or death. Patients who did not reach the event of interest during follow-up or those who were lost to follow-up were defined as censored. A 30% decline in eGFR or ESRD, whichever occurred first, was used as the definition of the primary endpoint.

### Immunohistochemical staining and Immunofluorescence staining of renal biopsies

Frozen renal biopsies from 104 patients with IgAN (from June 2019 to March 2021) were stained for C3c and galactose deficient IgA1 (Gd-IgA1) by immunofluorescence. Paraffin-embedded renal tissues from 10 patients with IgAN in group A were stained for FH, FHR-5, MBL, C4d, C3c, and membrane attack complex (MAC) by immunohistochemistry and immunofluorescence. Detail staining protocols were optimized for frozen renal biopsy tissue and formalin-fixed paraffin-embedded renal biopsy tissue with the following antibodies: mouse monoclonal anti-human FH antibody (Santa Cruz, United States), and rabbit polyclonal anti-human FHR-5 antibody (GeneTex, California, United States), mouse monoclonal anti-human MBL antibody (clone 3E7) (Hycult Biotech, United States), rabbit polyclonal anti-human C4d antibody (Bio-Rad, United States), mouse monoclonal anti-human C5b-9 antibody (Abcam, United States), rabbit polyclonal anti-human C3c (Dako, Glostrup, Denmark), rat monoclonal anti-human Gd-IgA1 antibody (KM55) (Immuno-Biological Laboratories, Japan) (11, 15). The method to detect colocalization of C3c and CD31 (biomarker of endothelial cells, Abcam, United States) were the same as previously described (15).

Immunofluorescence was scored using a fluorescence microscope (Nikon 80i, Japan). Two observers (Wei-yi Guo and Li-jun Sun), who were blinded to the clinical data, graded the staining intensity from anonymized sections as 0 (absent), 1+, 2+, or 3+. Sections that contained fewer than 2 glomeruli and 2 arterioles were excluded.

### Measurement of plasma galactose deficient IgA1

Plasma samples of 60 patients with IgAN (from June 2019 to March 2021) were collected on the day of biopsy and stored at −80^°^C. Levels of Gd-IgA1 was measured using a commercially available enzyme-linked immunosorbent assay test kit with KM55 (Immuno-Biological Laboratories, Japan) according to the manufacture’s protocol.

### Statistical analysis

Quantitative variables are presented as the means ± standard deviation (for normally distributed data) or medians with interquartile range (IQR) (for non-normally distributed data). Categorical data are summarized as absolute frequencies and percentages. For continuous variables, an independent-samples *t*-test was used when the data were normally distributed; for non-normally distributed data, the Mann-Whitney or Kruskal-Wallis test was performed. Categorical variables were compared using the χ^2^-test. The prognostic factors were identified by multivariate analysis with Cox regression. The results are expressed as hazard ratios (HRs) with 95% confidence intervals (CIs). SPSS 11.0 statistical software (SPSS, Chicago, IL, United States) was used for mean/median/percentage comparison and survival analysis.

## Results

### Clinical and histologic data

At the time of biopsy, there were 403/751 (53.7%) male patients, and the mean age was 38.17 ± 13.02 years. Prodromic infection and gross hematuria were found in 114 (15.2%) and 136 (18.2%) patients, respectively. At the time of renal biopsy, the median proteinuria value of the patients was 1.26 (0.60, 2.50) g/24 h, while the average estimated glomerular filtration rate (eGFR) was 82.31 (50.48, 107.67) ml/min/1.73 m^2^. Of our patients, 557/751 (74.2%) presented with hypertension. Histologically, mesangial hypercellularity (M1), endocapillary hypercellularity (E1), and segmental glomerulosclerosis (S1) were found in 300/751 (39.9%), 465/751 (61.9%) and 404 (53.8%) patients, respectively. For tubular atrophy and interstitial fibrosis (Oxford-T), 342/751 (45.5%), 252/751 (33.6%), and 157/751 (20.9%) of patients were scored as T0/T1/T2, respectively. For crescent lesions, 487/751 (64.8%), 203/751 (27.0%), and 61/751 (8.1%) of patients were scored as C0/C1/C2, respectively (Table 1).

**TABLE 1:** Demographic, clinical, and histologic characteristics of patients with IgAN.

	All patients *N* = 751	Group A *n* = 202	Group B *n* = 549	*P*-value
**Clinical features**				
Age (year)	38.17 ± 13.02	41.51 ± 12.73	36.93 ± 12.92	<0.001
Sex (male)	403 (53.7)	123 (60.9)	280 (51.0)	0.016
Prodromic infection (with, %)	114 (15.8)	21 (10.4)	93 (16.9)	0.027
Gross hematuria (with, %)	136 (18.2)	21 (10.4)	115 (21.0)	0.001
SBP (mmHg)	129 (119, 140)	130 (120,140)	128 (118, 140)	0.165
DBP (mmHg)	80 (74, 90)	82 (76, 90)	80 (74, 90)	0.131
Hypertension (with, %)	557 (74.2)	165 (81.7)	392 (71.4)	0.004
Malignant hypertension (with, %)	30 (4.0)	10 (5.0)	20 (3.6)	0.417
BMI (kg/m^2^)	25.15 ± 4.61	26.43 ± 4.66	24.68 ± 4.51	<0.001
Metabolic syndrome (with, %)	263 (35.0)	98 (48.5)	165 (30.1)	<0.001
**Laboratory measurements**				
UACR (mg/g)	328.33 (175.38, 647.46)	257.75 (148.22, 541.19)	366.07 (227.11, 728.48)	0.026
Initial proteinuria (g/d)	1.26 (0.60, 2.50)	1.00 (0.50, 2.40)	1.31 (0.62, 2.53)	0.033
Microscopic RBC < 3/HP (with, %)	101 (13.4)	54 (26.7)	47 (8.6)	<0.001
Urine osmotic pressure (mOsm/kg⋅H_2_O)	645.83 ± 189.03	668.89 ± 182.66	637.65 ± 190.81	0.066
HGB (g/L)	133.54 ± 21.76	138.75 ± 21.66	131.63 ± 21.50	<0.001
ALB (g/L)	38.08 ± 6.46	38.11 ± 7.74	38.07 ± 5.93	0.103
SCr (μmol/L)	90.90 (69.20, 136.00)	92.25 (72.73, 131.05)	90.30 (68.15, 140.30)	0.939
eGFR (ml/min per 1.73 m^2^)	82.31 (50.48, 107.67)	80.49 (55.17, 104.49)	82.46 (49.22, 109.19)	0.832
UA (μmol/L)	405.29 ± 113.07	398.54 ± 113.59	407.79 ± 112.88	0.496
sIgA (g/L)	2.98 ± 1.15	3.02 ± 1.18	2.97 ± 1.13	0.653
^#^Gd-IgA1 (μg/ml)	6.59 (4.14, 9.86)	6.24 (3.53, 12.94)	7.27 (4.88, 9.25)	0.687
sC3 (g/L)	1.09 (0.95, 1.25)	1.18 (0.98, 1.32)	1.07 (0.94, 1.22)	<0.001
sC4 (g/L)	0.25 ± 0.09	0.26 ± 0.14	0.24 ± 0.07	0.241
**Histologic features**				
Glomerulus IgA (1 + /2 + /3 + /4 +), *n* (%)	22 (2.9)/218 (29.0)/383 (50.1)/128 (17.0)	13 (6.4)/81 (40.1)/75 (37.1)/33 (16.3)	9 (1.6)/137 (25.0)/308 (56.1)/95 (17.3)	<0.001
^$^Glomerulus KM55 (0/1 + /2 + /3∼4 +), *n* (%)	25 (24.0)/30 (28.8)/37 (35.6)/12 (11.5)	18 (47.4)/7 (18.4)/9 (23.7)/4 (10.5)	7 (10.6)/23 (34.8)/28 (42.4)/8 (12.1)	0.003
Glomerulus C3 (0/1 + /2 + /3∼4 +), *n* (%)	65 (8.7)/64 (8.5)/263 (35.0)/359 (47.8)	29 (14.4)/28 (13.9)/70 (34.7)/75 (37.0)	36 (6.6)/36 (6.6)/193 (35.2)/284 (51.6)	<0.001
^$^Glomerulus C3c (0/1 + /2 + /3∼4 +), *n* (%)	7 (6.7)/10 (9.6)/50 (48.1)/37 (35.6)	4 (10.5)/5 (13.2)/20 (52.6)/9 (23.7)	3 (4.5)/5 (7.6)/30 (45.5)/28 (42.4)	0.027
^$^Artery C3c (0/1 + /2 + /3∼4 +), *n* (%)	55 (52.9)/17 (16.3)/23 (22.1)/9 (8.7)	17 (44.7)/3 (7.9)/14 (36.8)/4 (10.5)	38 (57.6)/14 (21.2)/9 (13.6)/5 (7.6)	0.048
Arteriolar lesions (mild/moderate/severe), *n* (%)	418 (48.3)/163 (18.8)/49 (5.7)	138 (68.3)/46 (22.8)/18 (8.9)	280 (51.0)/117 (21.3)/31 (5.6)	<0.001
**Oxford classification, *n* (%)**				
S1	404 (53.8)	65 (32.2)	339 (61.7)	<0.001
T1/T2	252 (33.6)/157 (20.9)	64 (31.7)/39 (19.3)	188 (34.2)/118 (21.5)	0.278
**Treatment**				
RASI, *n* (%)	519 (72.3)	155 (79.1)	364 (69.7)	0.013
Steroids or immunosuppressive agents, *n* (%)	206 (28.7)	31 (15.8)	175 (33.5)	<0.001

Group A, arteriolar lesions group; Group P, proliferative lesions group; BMI, body mass index; UACR, urinary albumin creatinine ratio; SCr, Serum creatinine; eGFR, estimate glomerular filtration rate; ALB, albumin; HBG, hemoglobin; C3, Complement C3; C3c, Complement C3c; C4, Complement C4; Oxford classification, segmental glomerulosclerosis/adhesion (S1: present), severity of tubular atrophy/interstitial fibrosis (T1: 26–50%, T2 > 50%); RASI: Renin angiotensin system inhibitor. ^$^There were 104 effective cases who were underwent immunofluorescence of C3c and Gd-IgA1 on renal biopsy from January 2019 to March 2021: group A (38 cases), group B (66 cases). ^#^There were 60 effective cases who were underwent plasma Gd-IgA1 by commercial ELISA kit from January 2019 to March 2021: group A (22 cases), group B (38 cases).

### Association of clinical, laboratory and pathologic parameters between group A and group B

There were significantly more IgAN patients in group A than in group B who experienced hypertension [165/202 (81.7%) vs. 392/549 (71.4%), *p* = 0.004, Table 1] and non-microscopic hematuria [54/202 (26.7%) vs. 47/549 (8.6%), *p* < 0.001, Table 1]. Compared with those in group B, fewer patients in group A experienced prodromic infection [21/202 (10.4%) vs. 93/549 (16.9%), *p* = 0.027, Table 1] and gross hematuria [21/202 (10.4%) vs. 115/549 (21.0%), *p* = 0.001, Table 1]. In addition, compared with those in group B, patients in group A presented with less initial proteinuria [1.00 (0.50, 2.40) g/day vs. 1.31 (0.62, 2.53) g/day, *p* = 0.033, Table 1], and percentages of segmental glomerulosclerosis/adhesion [S1: 65/202 (32.2%) vs. 339/549 (61.7%), *p* < 0.001, Table 1]. In terms of treatment, more patients in group A were treated with a renin-angiotensin system inhibitor (RASI) [155 (79.1%) vs. 364 (69.7%), *p* = 0.013, Table 1], and fewer patients in group A were treated with steroids or immunosuppressive agents [31 (15.8%) vs. 175 (33.5%), *p* < 0.001, Table 1] than patients in group B.

### Demographic, clinical, and histological characteristics of the subgroup of group A with IgA nephropathy based on the severity of arterial lesions

According to the severity of intrarenal arterial lesions, we divided the arteriolar lesions group into mild, moderate and severe subgroups. The severe subgroup presented with more severe left ventricular mass index (LVMI) [severe/moderate/mild subgroup: 105.50 (88.99, 129.26) g/m^2^ vs. 91.80 (70.67, 109.68) g/m^2^ vs. 80.33 (68.13, 101.03) g/m^2^, *p* = 0.002, Table 2], urine osmotic pressure (severe/moderate/mild subgroup: 536.28 ± 190.88 mOsm/kg⋅H_2_O vs. 642.20 ± 176.19 mOsm/kg⋅H_2_O vs. 697.60 ± 175.11 mOsm/kg⋅H_2_O, *p* < 0.001, Table 2), initial proteinuria [severe/moderate/mild subgroup: 2.59 (0.98, 4.05) g/day vs. 0.75 (0.44, 2.24) g/day vs. 0.98 (0.49, 2.36) g/day, *p* = 0.021, Table 2], and eGFR [severe/moderate/mild group: 47.11 (23.14, 72.59) ml/min/1.73 m^2^ vs. 68.28 (42.51, 87.38) ml/min/1.73 m^2^ vs. 91.42 (63.52, 111.49) ml/min/1.73 m^2^, *p* < 0.001, Table 2] and a high percentage of Oxford T lesions [severe/moderate/mild subgroup: T1/T2, 7 (38.9%)/10 (55.6%) vs. 19 (41.3%)/11 (23.9%) vs. 38 (27.5%)/18 (13.0%), *p* < 0.001, Table 2].

**TABLE 2:** Demographic, clinical, and histological characteristics of the subgroup of the arteriolar lesions group with IgAN based on the severity of arterial lesions. (*n* = 202).

	Mild subgroup *n* = 138	Moderate subgroup *n* = 46	Severe subgroup *n* = 18	*P*-value
**Clinical features**				
Age (year)	41.59 ± 12.41	41.65 ± 14.28	40.56 ± 11.52	0.936
Sex (male)	76 (55.1)	33 (71.7)	14 (77.8)	0.014
Prodromic infection (with, %)	12 (8.7)	4 (8.7)	5 (27.8)	0.052
Gross hematuria (with,%)	14 (10.1)	6 (13.0)	1 (5.6)	0.852
SBP (mmHg)	129 (118,140)	130 (121, 141)	130 (122, 143)	0.207
DBP (mmHg)	82 (75, 90)	80 (77, 90)	85 (78, 100)	0.385
Hypertension (with, %)	108 (78.3)	40 (87.0)	17 (94.4)	0.049
Malignant hypertension (with, %)	1 (0.7)	4 (8.7)	5 (27.8)	<0.001
Metabolic syndrome (with, %)	67 (48.6)	18 (39.1)	13 (72.2)	0.360
LVMI (g/m^2^)	80.33 (68.13, 101.03)	91.80 (70.67, 109.68)	105.50 (88.99, 129.26)	0.002
**Laboratory measurements**				
UACR (mg/g)	192.68 (111.28, 408.50)	255.57 (209.14, 340.28)	735.77 (253.38, 922.21)	0.087
Initial proteinuria (g/d)	0.98 (0.49, 2.36)	0.75 (0.44, 2.24)	2.59 (0.98, 4.05)	0.021
Microscopic RBC < 3/HP (with, %)	32 (23.2)	15 (32.6)	7 (38.9)	0.083
Urine osmotic pressure (mOsm/kg⋅H_2_O)	697.60 ± 175.11	642.20 ± 176.19	536.28 ± 190.88	0.001
HGB (g/L)	138.90 ± 20.98	139.00 ± 22.45	136.94 ± 25.73	0.934
ALB (g/L)	37.46 ± 8.24	39.20 ± 6.93	40.23 ± 4.87	0.253
SCr (μmol/L)	86.45 (63.98, 110.93)	118.20 (84.33, 143.83)	141.20 (103.70, 236.58)	< 0.001
eGFR (ml/min per 1.73 m^2^)	91.42 (63.52, 111.49)	68.28 (42.51, 87.38)	47.11 (23.14, 72.59)	< 0.001
UA (μmol/L)	382.43 ± 111.83	422.72 ± 111.54	460.25 ± 105.47	0.003
sIgA (g/L)	3.15 ± 1.26	2.92 ± 0.96	2.32 ± 0.64	0.013
sC3 (g/L)	1.18 (0.99, 1.32)	1.15 (0.98, 1.32)	1.17 (0.92, 1.35)	0.960
sC4 (g/L)	0.25 ± 0.09	0.30 ± 0.21	0.28 ± 0.10	0.349
**Histologic features**				
Glomerulus IgA (1 + /2 + /3 + /4 +), *n* (%)	8 (5.8)/52 (37.7)/52 (37.7)/26 (18.8)	4 (8.7)/18 (39.1)/18 (39.1)/6 (13.0)	1 (5.6)/11 (61.1)/5 (27.8)/1 (5.6)	0.068
Glomerulus C3 (0/1 + /2 + /3 + ∼4 +), *n* (%)	20 (14.5)/17 (12.3)/51 (37.0)/50 (36.2)	7 (15.2)/5 (10.9)/15 (32.6)/19 (41.3)	2 (11.1)/6 (33.3)/4 (22.2)/6 (33.4)	0.704
^$^Glomerulus C3c (0/1 + /2 + /3 + ∼4 +), *n* (%)	3 (13.0)/4 (17.4)/11 (47.8)/5 (21.7)	1 (10.0)/0 (0.0)/7 (70.0)/2 (20.0)	0 (0.0)/1 (20.0)/2 (40.0)/2 (40.0)	0.298
^$^Artery C3c (0/1 + /2 + /3 +), *n* (%)	14 (60.9)/1 (4.3)/7 (30.4)/1 (4.3)	2 (20.0)/1 (10.0)/5 (50.0)/2 (20.0)	1 (20.0)/1 (20.0)/2 (40.0)/1 (20.0)	0.036
**Oxford classification, *n* (%)**				
S1	39 (28.3)	19 (41.3)	7 (38.9)	0.125
T1/T2	38 (27.5)/18 (13.0)	19 (41.3)/11 (23.9)	7 (38.9)/10 (55.6)	<0.001
**Treatment**				
RASI, *n* (%)	105 (77.8)	36 (83.7)	14 (77.8)	0.682
Steroids or immunosuppressive agents, *n* (%)	29 (21.5)	1 (2.3)	1 (5.6)	0.004

BMI, body mass index; UACR, urinary albumin creatinine ratio; SCr, Serum creatinine; eGFR, estimate glomerular filtration rate; ALB, albumin; HBG, hemoglobin; C3, Complement C3; C3c, Complement C3c; C4, Complement C4; LVMI, left ventricular mass index; Oxford classification: segmental glomerulosclerosis/adhesion (S1: present), severity of tubular atrophy/interstitial fibrosis (T1: 26–50%, T2 > 50%); RASI, Renin angiotensin system inhibitor. ^$^There were 104 effective cases who were underwent immunofluorescence of C3c on renal biopsy from January 2019 to March 2021. Among them, there were 38 cases in arteriole lesions group: Mild subgroup (23 cases), Moderate subgroup (10 cases) and severe subgroup (5 cases).

### The hypertension subgroup and the non-hypertension subgroup in patients with IgA nephropathy in group A

In the arteriolar lesions group, patients without hypertension had a higher eGFR [97.38 (75.81, 120.22) ml/min/1.73 m^2^ vs. 78.10 (49.38, 102.87) ml/min/1.73 m^2^, *p* < 0.001] and fewer arteriolar lesions [mild/moderated/severe lesions: 30 (81.1%)/6 (16.2%)/1 (2.7%) vs. 108 (65.5%)/40 (24.2%)/17 (10.3%), *p* = 0.049, Table 3]. Interestingly, the hypertension and non-hypertension subgroups of IgAN patients in the arteriolar lesion group histological exhibited no differences in the presence of lesions such as Oxford S lesions and T lesions and in treatment with a RASI, steroids or immunosuppressive agents.

**TABLE 3:** Demographic, clinical, and histologic characteristics of hypertension and non-hypertension subgroup in arteriolar lesions group of IgAN patients.

	Non-hypertension subgroup *n* = 37	Hypertension subgroup *n* = 165	*P*-value
**Clinical features**			
Age (yr)	38.08 ± 14.49	42.28 ± 12.21	0.069
Sex (male)	21 (56.8)	102 (61.8)	0.569
Prodromic infection (with, %)	4 (10.8)	17 (10.3)	0.927
Gross hematuria (with, %)	7 (18.9)	14 (8.5)	0.060
SBP (mmHg)	117 (110,123)	130 (122, 144)	<0.001
DBP (mmHg)	76 (70, 80)	85 (79, 95)	<0.001
Malignant hypertension (with, %)	0 (0.0)	10 (6.1)	0.125
BMI (kg/m^2^)	24.17 ± 3.88	26.94 ± 4.67	0.002
Metabolic syndrome (with, %)	7 (18.9)	91 (55.2)	<0.001
LVMI (g/m^2^)	84.82 (71.95, 100.76)	86.58 (69.87, 108.44)	0.755
**Laboratory measurements**			
UACR (mg/g)	143.16 (40.21, 208.05)	292.31 (162.79, 587.65)	0.023
Initial proteinuria (g/d)	0.90 (0.50, 3.78)	1.00 (0.50, 2.40)	0.757
Microscopic RBC < 3/HP (with, %)	7 (18.9)	47 (28.5)	0.235
Urine osmotic pressure (mOsm/kg⋅H_2_O)	712.34 ± 159.72	658.95 ± 186.57	0.119
HGB (g/L)	144.32 ± 16.49	137.50 ± 22.51	0.156
ALB (g/L)	35.41 ± 9.86	38.71 ± 7.08	0.032
SCr (μmol/L)	84.20 (60.30, 102.05)	98.60 (75.55,135.80)	0.003
eGFR (ml/min per 1.73 m^2^)	97.38 (75.81, 120.22)	78.10 (49.38, 102.87)	0.001
UA (μmol/L)	365.66 ± 104.45	405.92 ± 114.54	0.101
sIgA (g/L)	3.17 ± 1.05	2.99 ± 1.20	0.244
sC3 (g/L)	1.07 (0.91, 1.26)	1.19 (1.00, 1.32)	0.032
sC4 (g/L)	0.28 ± 0.26	0.26 ± 0.10	0.332
**Histologic features**			
Glomerulus IgA (1 + /2 + /3 + /4 +), *n* (%)	3 (8.1)/12 (32.4)/14 (37.8)/8 (21.6)	10 (6.1)/69 (41.8)/61 (37.0)/25 (15.2)	0.650
Glomerulus C3 (0/1 + /2 + /3 + ∼4 +), *n* (%)	2 (5.4)/8 (21.6)/8 (21.6)/19 (51.4)	27 (16.4)/20 (12.1)/62 (37.6)/56 (33.9)	0.202
^$^Glomerulus C3c (0/1 + /2 + /3 + ∼4 +), *n* (%)	1 (25.0)/2 (50.0)/0 (0.0)/1 (25.0)	3 (8.8)/3 (8.8)/20 (58.8)/8 (23.5)	0.127
^$^Artery C3c (0/1 + /2 + /3 + ∼4 +), *n* (%)	3 (75.0)/0 (0.0)/1 (25.0)/0 (0.0)	14 (41.2)/3 (8.8)/13 (38.2)/4 (11.8)	0.233
Arteriolar lesions (mild/moderate/severe), *n* (%)	30 (81.1)/6 (16.2)/1 (2.7)	108 (65.5)/40 (24.2)/17 (10.3)	0.049
Oxford classification, *n* (%)			
S1	12 (32.4)	53 (32.1)	0.971
T1/T2	12 (32.4)/4 (10.8)	52 (31.5)/35 (21.2)	0.157
**Treatment**			
RASI, *n* (%)	23 (67.6)	132 (81.5)	0.071
Steroids or immunosuppressive agents, *n* (%)	7 (20.6)	24 (14.8)	0.402

BMI, body mass index; UACR, urinary albumin creatinine ratio; SCr, Serum creatinine; eGFR, estimate glomerular filtration rate; ALB, albumin; HBG, hemoglobin; C3, Complement C3; C3c, Complement C3c; C4, Complement C4; LVMI, left ventricular mass index; Oxford classification, segmental glomerulosclerosis/adhesion (S1: present), severity of tubular atrophy/interstitial fibrosis (T1: 26–50%, T2 > 50%); RASI, Renin angiotensin system inhibitor. ^$^There were 104 effective cases who were underwent immunofluorescence of C3c on renal biopsy from January 2019 to March 2021. Among them, there were 38 cases in arteriole lesions group: Non-hypertension subgroup (4 cases), Hypertension subgroup (34 cases).

### Patients with IgA nephropathy in group A showed no better renal survival in progression than those in group B

The clinical, laboratory and pathologic parameters between group A and group B with regular follow-up were showed in Table 4. During follow-up, 18 patients (24.0%) in group A and 67 patients (32.2%) in group B reached the composite endpoint (*p* = 0.184). Among these patients, 11 patients (16.2%) in group A and 46 patients (24.6%) in group B showed a 30% decline in eGFR (*p* = 0.153). Moreover, end-stage renal disease occurred in 5 patients (5.2%) in group A and 33 patients (11.4%) in group B (*p* = 0.075).

**TABLE 4:** Demographic, clinical, and histologic characteristics of patients with IgAN with follow-up. (*n* = 255).

	Group A *n* = 68	Group B *n* = 187	*P*-value
**Clinical features**			
Age (yr)	42.68 ± 12.37	35.93 ± 12.66	<0.001
Sex (male)	42 (61.8)	90 (48.1)	0.054
Prodromic infection (with, %)	7 (10.3)	21 (11.2)	0.833
Gross hematuria (with, %)	9 (13.2)	39 (21.0)	0.163
SBP (mmHg)	130 (120,139)	128 (118, 140)	0.787
DBP (mmHg)	82 (77, 90)	80 (75, 90)	0.497
Hypertension (with, %)	54 (79.4)	138 (73.8)	0.358
Malignant hypertension (with, %)	3 (4.4)	6 (3.2)	0.645
BMI (kg/m^2^)	26.88 ± 4.58	24.33 ± 3.99	<0.001
Metabolic syndrome (with, %)	36 (52.9)	52 (27.8)	<0.001
LVMI (g/m^2^)	82.95 (70.80, 107.13)	88.21 (74.66, 99.86)	0.886
**Laboratory measurements**			
UACR (mg/g)	255.57 (81.03, 576.57)	523.57 (307.33, 1046.27)	0.026
Initial proteinuria (g/d)	0.88 (0.49, 2.37)	1.40 (0.74, 2.62)	0.010
Microscopic RBC < 3/HP (with, %)	15 (22.1)	17 (9.1)	<0.001
Urine osmotic pressure (mOsm/kg⋅H_2_O)	672.43 ± 189.00	631.78 ± 180.28	0.120
HGB (g/L)	142.19 ± 21.09	130.90 ± 20.36	<0.001
ALB (g/L)	38.86 ± 8.31	37.90 ± 5.94	0.014
SCr (μmol/L)	85.05 (69.33, 112.13)	91.00 (69.70, 151.00)	0.107
eGFR (ml/min per 1.73 m^2^)	88.94 (63.15, 105.26)	80.86 (42.36, 110.31)	0.234
UA (μmol/L)	394.80 ± 95.99	413.10 ± 119.80	0.385
sIgA (g/L)	3.14 ± 1.20	2.91 ± 1.07	0.196
sC3 (g/L)	1.20 ± 0.20	1.09 ± 0.21	<0.001
sC4 (g/L)	0.24 (0.19, 0.28)	0.23 (0.20, 0.27)	0.847
**Histologic features**			
Glomerulus IgA (1 + /2 + /3 + /4 +), *n* (%)	5 (7.4)/33 (48.5)/23 (33.8)/7 (10.3)	3 (1.6)/47 (25.1)/105 (56.1)/32 (17.1)	<0.001
Glomerulus C3 (0/1 + /2 + /3 + ∼4 +), *n* (%)	12 (17.6)/12 (17.6)/20 (29.4)/24 (35.4)	11 (5.9)/14 (7.5)/67 (35.8)/95 (50.8)	0.001
^$^Glomerulus C3c (0/1 + /2 + /3 + ∼4 +), *n* (%)	2 (15.4)/3 (23.1)/6 (46.2)/2 (15.4)	0 (0.0)/1 (6.7)/6 (40.0)/8 (53.3)	0.011
^$^Artery C3c (0/1 + /2 + /3 + ∼4 +), *n* (%)	6 (46.2)/1 (7.7)/5 (38.5)/1 (7.7)	8 (53.3)/4 (26.7)/2 (13.3)/1 (6.7)	0.379
Arteriolar lesions (mild/moderate/severe), *n* (%)	46 (67.6)/14 (20.6)/8 (11.8)	98 (52.4)/48 (25.7)/9 (4.8)	<0.001
Oxford classification, *n* (%)			
S1	18 (26.5)	121 (64.7)	<0.001
T1/T2	25 (36.8)/9 (13.2)	63 (33.7)/47 (25.1)	0.061
**Treatment**			
RASI, *n* (%)	57 (86.4)	120 (67.0)	0.003
Steroids or immunosuppressive agents, *n* (%)	6 (9.1)	65 (36.3)	<0.001

Group A, arteriolar lesions group; Group B, proliferative lesions group; BMI, body mass index; UACR, urinary albumin creatinine ratio; SCr, Serum creatinine; eGFR, estimate glomerular filtration rate; ALB, albumin; HBG, hemoglobin; C3, Complement C3; C3c, Complement C3c; C4, Complement C4; LVMI, left ventricular mass index; Oxford classification: segmental glomerulosclerosis/adhesion (S1: present), severity of tubular atrophy/interstitial fibrosis (T1: 26–50%, T2 > 50%); RASI, Renin angiotensin system inhibitor. ^$^There were 28 effective cases who were underwent immunofluorescence of C3c on renal biopsy from January 2019 to March 2021. Among them, 13 cases were in group A and 15 cases in group B were regularly followed for at least 1 year.

**FIGURE 4:**
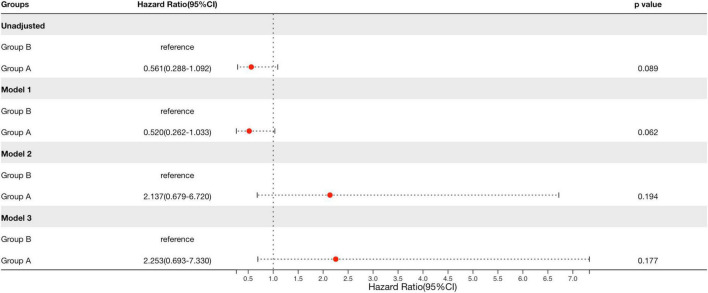
Cox regression to compare the prognosis between the arteriolar lesions group and proliferative lesions group in patients with IgAN. Group A, arteriolar lesion group; Group B, proliferative lesion group. Model 1 was adjusted for sex and age. Model 2 was adjusted for covariates in model 1 plus eGFR, proteinuria, history of hypertension (yes or no), and Oxford classification-MESTC. The latter five variables were analyzed as categorical data. Model 3 was adjusted for covariates in model 2 plus RASI and steroids or other immunosuppressive agents use (yes or no). Renal survival in group A was not better than that in group B after adjustment for multiple clinical and histological risk factors, as well as therapy.

We used the Cox proportional hazards model to compare prognosis between group A and group B. After adjustment for multiple clinical and histological risk factors, as well as steroids/immunosuppressive therapy, patients with group A showed no significantly risks of reaching the composite outcome compared with group B (HR = 2.253, 95% CI: 0.693–7.330, *p* = 0.194, Table 5 and Figure 4), which revealed that renal survival in group A was not better than that in group B.

**TABLE 5:** Cox regression to compare the prognosis between the arteriolar lesion group and proliferative lesion group.

	Unadjusted	*P*-value	Model 1^a^	*P*-value	Model 2^b^	*P*-value	Model 3^c^	*P*-value
Group B Group A	Reference 0.561 (0.288–1.092)	0.089	Reference 0.520 (0.262–1.033)	0.062	Reference 2.137 (0.679–6.720)	0.194	Reference 2.253 (0.693–7.330)	0.177

Composite end point was defined as a 30% decline in eGFR or end stage renal disease which comes first. Group A, arteriolar lesions group; Group B, proliferative lesions group; Oxford classification: mesangial hypercellularity score (M1 > 0.5), the presence of endocapillary proliferation (E1: present), segmental glomerulosclerosis/adhesion (S1: present), severity of tubular atrophy/interstitial fibrosis (T1: 26–50%, T2 > 50%) and presence of crescent (C1: 1–25%, C2: 26–100%). RASI, Renin angiotensin system inhibitor.

^*a*^Model 1 was adjusted for sex and age.

^*b*^Model 2 was adjusted for covariates in model 1 plus eGFR, proteinuria, history of hypertension (yes or no), and Oxford classification-MESTC. The latter five variables were analyzed as categorical data.

^*c*^Model 3 was adjusted for covariates in model 2 plus RASI and steroids or other immunosuppressive agents use (yes or no).

### Complement components deposited in the intrarenal arterioles in patients with IgA nephropathy

**FIGURE 5:**
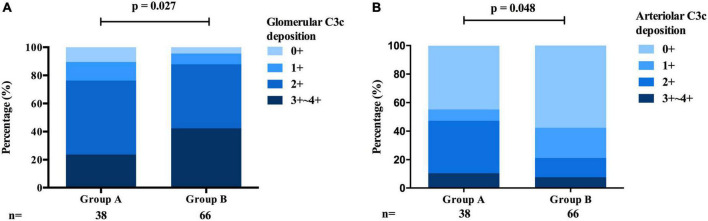
Comparison of the deposition of C3c between the arteriolar lesions group and the proliferative lesions group of patients with IgAN. **(A)** The intensity of C3c deposition in the glomerular mesangial and capillary areas was more severe in the proliferative lesions group than in the arteriolar lesions group. **(B)** The intensity of C3c deposition in intrarenal arterioles was more severe in the arteriolar lesions group than in the proliferative lesions group.

**FIGURE 6:**
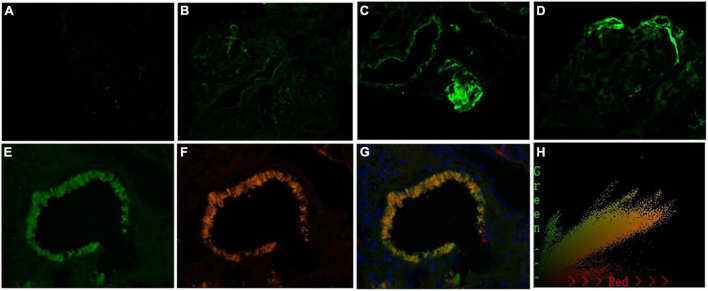
Representative images of immunofluorescence staining for C3c in arteries grade 0–3 +, and the colocalization of C3c and CD31 in patients with immunoglobulin A nephropathy (IgAN). Granular positive staining for C3c by immunofluorescence along intrarenal arterioles in patients with IgAN. **(A)** Negative. **(B)** 1 + intensity. **(C)** 2 + intensity. **(D)** 3 + intensity. (Original magnification × 200). **(E)** Granular positive staining for C3c, and **(F)** Granular positive staining for CD31 (biomarker of endothelial cells) by immunofluorescence along intrarenal arterioles. **(G)** C3c and CD31 colocalized along the intrarenal arterioles. **(H)** The corresponding 2-dimensional fluorograms have been included to confirm the degree of colocalization (Pearson correlation = 0.928389, overlap coefficient = 0.928594). (Original magnification ×400).

Interestingly, patients in group A exhibited a lower intensity of C3 [0/1 + /2 + /3 + ∼4 + : 17 (44.7%)/3 (7.9%)/14 (36.8%)/4 (10.5%) vs. 38 (57.6%)/14 (21.2%)/9 (13.6%)/5 (7.6%), *p* = 0.027, Table 1 and Figure 5A] deposition in the glomerular mesangial and capillary areas than those in group B. In addition, the serum C3 level was higher in group A than in group B [1.18 (0.98, 1.32) mmol/L vs. 1.07 (0.94, 1.22) mmol/L, *p* < 0.001, Table 1], indicating that complement activation in both the circulation and glomeruli was more severe in group B than in group A. Moreover, patients in group A exhibited a higher intensity of C3c deposition [0/1 + /2 + /3 + ∼4 + : 17 (44.7%)/3 (7.9%)/14 (36.8%)/4 (10.5%) vs. 38 (57.6%)/14 (21.2%)/9 (13.6%)/5 (7.6%), *p* = 0.048, Table 1 and Figure 5B] in intrarenal arterioles than those in group B, indicating that complement activation was more severe in intrarenal arterioles in group A than in group B. In group A, as the severity of intrarenal arteriolar lesions worsened, the intensity of C3c deposition in intrarenal arterioles increased [0/1 + /2 + /3 + ∼4 + : mild subgroup: 14 (60.9%)/1 (4.3%)/7 (30.4%)/1 (4.3%) vs. moderate subgroup: 2 (20.0%)/1 (10.0%)/5 (50.0%)/2 (20.0%) vs. severe subgroup: 1 (20.0%)/1 (20.0%)/2 (40.0%)/1 (20.0%), *p* = 0.036, Table 2], and patients with or without hypertension showed no differ in the intensity of C3c deposition in intrarenal arterioles [0/1 + /2 + /3 + ∼4 + : hypertension subgroup: 14 (41.2%)/3 (8.8%)/13 (38.2%)/4 (11.8%) vs. non-hypertension subgroup: 3 (75%)/0 (0.0%)/1 (25.0%)/0 (0.0%), *p* = 0.233, Table 3]. Figures 6A–D shows representative images of immunofluorescence staining for C3c deposition in intrarenal arterioles (grades 0–3 +) in patients with IgAN. C3c and CD31 (biomarker of endothelial cells) co-deposited in intrarenal arterioles area in patients with IgAN with intrarenal arteriolar lesions. 2-dimensional (2D) fluorograms were used to analyze colocalization by Image-Pro Plus software (Media Cybernetics, Rockville, MD) (Pearson correlation = 0.928389, overlap coefficient = 0.928594) (Figures 6E–H).

**FIGURE 7:**
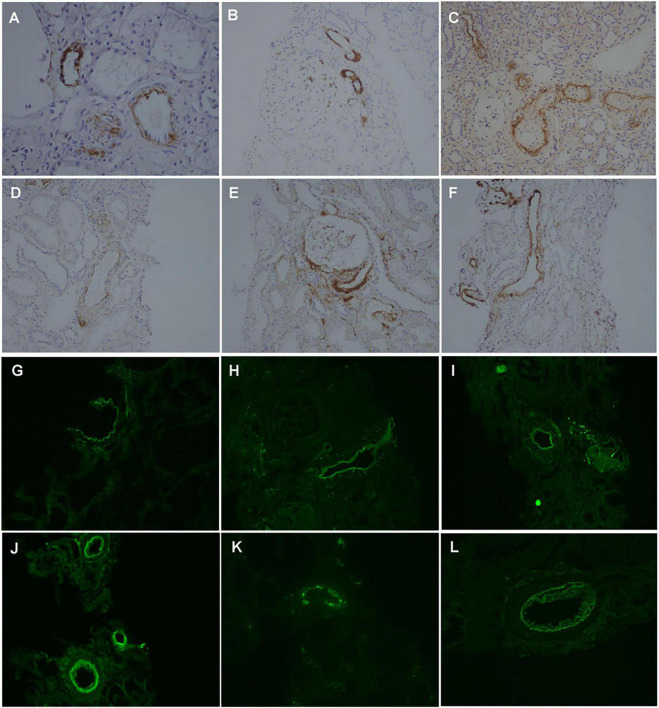
Positive staining for MBL **(A,G)**, C4d **(B,H)**, FH **(C,I)**, FHR5 **(D,J)**, C3c **(E,K)**, and MAC **(F,L)** by immunohistochemistry and immunofluorescence, respectively, along intrarenal arterioles in the arteriolar lesions group of IgAN. (Original magnification ×200).

Furthermore, renal biopsies were stained with immunohistochemistry and immunofluorescence for complement components such as MBL, C4d, FH, and FHR5 and MAC. MBL (Figures 7A,G), C4d (Figures 7B,H), FH (Figures 7C,I), FHR5 (Figures 7D,J), C3c (Figures 7E,K), and MAC (Figures 7F,L) were found to be deposited in the intrarenal arterioles in group A of IgAN.

### Plasma galactose deficient IgA1 and Glomerular galactose deficient IgA1 between group A and group B

**FIGURE 8:**
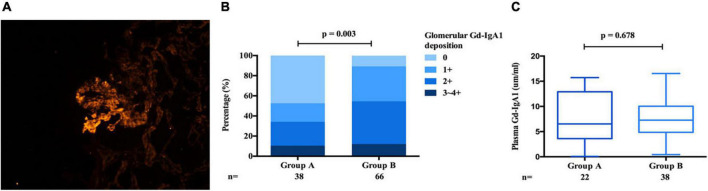
Gd-IgA1 in IgAN. Granular Gd-IgA1 (KM55) by immunofluorescence along the glomerular mesangial and capillary area in patients with IgAN **(A)**. Gd-IgA1 mainly deposited in glomerulus and the intensity of Gd-IgA1 deposition in group B was significantly stronger than that of group A **(B)**. Plasma Gd-IgA1 shows no differ in group A and group B **(C)**.

Gd-IgA1 mainly deposited in glomerulus and rarely deposited in the renal intrarenal arterioles (Figure 8A). Compared with IgAN patients in group B, patients in group A. In histological features, patients in group A exhibited a lower intensity of IgA [1 + /2 + /3 + /4 + : 13 (6.4%)/81 (40.1%)/75 (37.1%)/33 (16.3%) vs. 9 (1.6%)/137 (25.0%)/308 (56.1%)/95 (17.3%), *p* < 0.001, Table 1] and glomerular Gd-IgA1 [0/1 + /2 + /3∼4 + : 18 (47.4%)/7 (18.4%)/9 (23.7%)/4 (10.5%) vs. 7 (10.6%)/23 (34.8%)/28 (42.4%)/8 (12.1%), *p* = 0.003, Table 1 and Figure 8C] deposition in the glomerular mesangial and capillary areas than those in group B. There is no significant difference in serum IgA (group A: 3.02 ± 1.18 g/L vs. group B: 2.97 ± 1.13 g/L, *p* = 0.653, Table 1) and plasma Gd-IgA1 [group A: 6.24 (3.53, 12.94) g/L vs. group B: 7.27 (4.88, 9.25) g/L, *p* = 0.687, Table 1 and Figure 8C] between group A and group B.

## Discussion

Some patients with IgAN present atypical clinical manifestations, that is, present without protruding hematuria, with or without hypertension. The renal biopsy pathology of these patients shows predominant intrarenal arteriolar lesions. The renal function of patients with IgAN with this phenotype can deteriorate rapidly. This study focused on studying the clinical and pathological characteristics and prognosis of patients with this phenotype of IgAN and preliminarily explored whether complement is overactivated in this vascular disease.

In the present study, we found that compared with those in the proliferative lesions group, more IgAN patients in the arteriolar lesions group experienced hypertension, and fewer patients in the arteriolar lesions group experienced gross hematuria and microscopic hematuria. In addition, compared with those in the proliferative lesions group, patients in the arteriolar lesions group presented with less initial proteinuria. We also found that the renal prognosis of IgAN with only arteriolar lesions was not better than that of IgAN with proliferative lesions. A retrospective study found that the prevalence of intrarenal arterial lesions was up to 72.2% in patients with IgAN and that the presence of vascular lesions was associated with a reduced eGFR and poorer renal outcomes (6). Wu et al. (8) analyzed the intrarenal arterial lesions in 1005 Chinese patients with IgAN and found that the prevalence of intrarenal small artery and arteriolar lesions was 54.6%. Moreover, the severity of small arterial-arteriolar lesions was linked to adverse renal outcomes. Similarly, Nasri and Mubarak (25) found that 47.8% of IgAN patients presented with arteriosclerosis and 41.9% with arterial intimal fibrosis, and these arteriolar lesions were related to elevated levels of serum creatinine and proteinuria. However, the Oxford multicenter cohort study indicated that arteriolar lesions are not an independent risk factor that affects the prognosis of IgAN. They found that 40% of patients presented with arteriolar lesions, which was strongly correlated with the level of blood pressure and eGFR but not with proteinuria severity, and there was no significant correlation between arterial lesion severity and renal prognosis (26). Previous studies on vascular lesions in IgAN mostly focused on those patients with arteriolar lesions accompanied by proliferative lesions. However, our study mainly investigated IgAN with intrarenal arteriolar sclerosis lesions and rarely with glomerular proliferative lesions. We found that the renal prognosis of the arteriolar lesions group was not better than that of the proliferative lesions group.

Previous studies have shown that blood pressure is an independent factor that affects arteriolar injury in IgAN (6). However, our research and previous studies have shown that patients with IgAN, even those with normal blood pressure present with renal arteriolar sclerosis (8). Thus, we speculate that, apart from hypertension, other factors are involved in the pathogenesis and induce a poor renal prognosis in this phenotype of IgAN. In this study, we found that the positive rate of complement C3c deposition on the intrarenal arteriolar wall was 50/116 (43.1%). In the arteriolar lesions group, the intensity of C3c deposition on the walls of intrarenal arterioles was significantly stronger than that in the proliferative lesions group. Complement activation can generally occur through three different pathways (Figure 1). In this study, we found that complement components such as MBL (representing lectin pathway), C4d (the classical and lectin pathways), FH, and FHR5 (the alternative pathway), and MAC (the terminal pathway) are deposited on the walls of injured intrarenal arterioles, which suggests that the abnormal deposition of the above complement components on the intrarenal arteriolar wall may be involved in the pathogenesis of arteriole damage. Faria et al. (19) found that arteriolar C4d deposition was identified in 21/126 (16.7%) IgAN patients and that arteriolar C4d was a potential biomarker for disease progression in IgAN. Importantly, we also found that both hypertensive and non-hypertensive patients in the arteriolar lesions group showed no difference in the intensity of complement C3c deposition on the arterial wall. We speculated that the abnormal activation of the complement system may be involved in the pathogenesis of arteriolar damage, which might affect the prognosis of this clinical phenotype of IgAN.

Our study showed that, in addition to the deposition of complement MBL, C4d, C3c, and MAC on the damaged arteriolar wall, complement FH and FHR5 are also deposited on the wall of injured arterioles. FH is a negative regulatory protein in the alternative pathway that prevents overactivation of complement (27). FHR5 is a natural antagonist of FH and deregulates FH to overactivate the complement system (28, 29). Our previous research found that glomerular staining for FHR5 was observed in 57% of predominantly mesangial pattern with IgAN in biopsy specimens (11). The glomerular intensity of FHR5 deposition could indicate the severity of histologic lesions in IgAN (11, 30). Previous studies have also shown that FHR5 is deposited on the surface of vascular endothelial cells (31). We also speculate that FHR5 and FH are deposited in the endothelial cells of renal intrarenal arterioles and that then FHR5 competitively antagonizes the negative regulation of complement activation by FH, thus overactivating the complement system (32). Then, MACs form and damage endothelial cells, leading to arteriolar damage. The specific mechanism needs further exploration.

Gd-IgA1 is a pivotal molecule in the pathogenesis of IgAN (33–37). We found that plasma Gd-IgA1 shows no differ in proliferative lesions group and the arteriolar lesion group. Gd-IgA1 mainly deposited in glomerulus and the intensity of Gd-IgA1 deposition in the proliferative lesions group was significantly stronger than that of the arteriolar lesions group. However, Gd-IgA1 barely deposited in the renal intrarenal arterioles. We speculate that the damage of Gd-IgA1 to kidney might focus on glomerulus rather than intrarenal arterioles. We also found that complement C3c colocalized with CD31, a biomarker of endothelial cells, in intrarenal arterioles. We speculate that complement overactivation may cause the damage to intrarenal arterioles by injuring endothelial cells. In previous studies on atherosclerosis of the large and medium vasculature, the terminal complement complex C5b-9 affects atherogenesis by propelling vascular smooth muscle cell proliferation and migration, stimulating endothelial proliferation, and promoting vascular lesions formation (38). However, the specific mechanism of the complement system involved in intrarenal arteriole injury in this clinical phenotype of IgAN, which deserves further exploration.

We found that abnormal activation of complement may be involved in the pathogenesis of arteriolar damage in patients with IgAN, leading to a poor renal prognosis. We hope that our research might provide a theoretical basis for the intervention of complement activation in this clinical phenotype of IgAN in the future.

## Data availability statement

The raw data supporting the conclusions of this article will be made available by the authors, without undue reservation.

## Ethics statement

The studies involving human participants were reviewed and approved by the Ethics Committees of Beijing Anzhen Hospital. The patients/participants provided their written informed consent to participate in this study.

## Author contributions

W-YG and HC made substantial contributions to the study concept and design. W-YG drafted the manuscript. HC critically revised the manuscript, supervised the entire study, and gave final approval to the article. W-YG, X-PA, and H-RD performed the experiments. W-YG and L-JS reviewed kidney biopsies from all patients and graded the staining intensity from anonymized sections. W-YG, X-PA, and NY conducted statistical analyses. NY, X-YX, G-QW, Z-RZ, and W-RC treated the patients and collected the primary data. All authors read and approved the final manuscript.
